# NSF DARE—transforming modeling in neurorehabilitation: perspectives and opportunities from US funding agencies

**DOI:** 10.1186/s12984-024-01308-x

**Published:** 2024-02-03

**Authors:** Grace M. Hwang, Jonathan Kulwatno, Theresa H. Cruz, Daofen Chen, Toyin Ajisafe, Joseph D. Monaco, Ralph Nitkin, Stephanie M. George, Carol Lucas, Steven M. Zehnder, Lucy T. Zhang

**Affiliations:** 1 National Institute of Neurological Disorders and Stroke, National Institutes of Health, Rockville, MD 20852 USA; 2 Directorate for Engineering, National Science Foundation, 2415 Eisenhower Avenue, Alexandria, VA 22314 USA; 3 Eunice Kennedy Shriver National Institute of Child Health and Human Development, National Institutes of Health, Bethesda, MD 20817 USA

**Keywords:** Neurorehabilitation, Computational modeling, Digital twin, Federal government, Workforce, Computational neuroscience, Conference

## Abstract

In recognition of the importance and timeliness of computational models for accelerating progress in neurorehabilitation, the U.S. National Science Foundation (NSF) and the National Institutes of Health (NIH) sponsored a conference in March 2023 at the University of Southern California that drew global participation from engineers, scientists, clinicians, and trainees. This commentary highlights promising applications of computational models to understand neurorehabilitation (“Using computational models to understand complex mechanisms in neurorehabilitation” section), improve rehabilitation care in the context of digital twin frameworks (“Using computational models to improve delivery and implementation of rehabilitation care” section), and empower future interdisciplinary workforces to deliver higher-quality clinical care using computational models (“Using computational models in neurorehabilitation requires an interdisciplinary workforce” section). The authors describe near-term gaps and opportunities, all of which encourage interdisciplinary team science. Four major opportunities were identified including (1) deciphering the relationship between engineering figures of merit—a term commonly used by engineers to objectively quantify the performance of a device, system, method, or material relative to existing state of the art—and clinical outcome measures, (2) validating computational models from engineering and patient perspectives, (3) creating and curating datasets that are made publicly accessible, and (4) developing new transdisciplinary frameworks, theories, and models that incorporate the complexities of the nervous and musculoskeletal systems. This commentary summarizes U.S. funding opportunities by two Federal agencies that support computational research in neurorehabilitation. The NSF has funding programs that support high-risk/high-reward research proposals on computational methods in neurorehabilitation informed by theory- and data-driven approaches. The NIH supports the development of new interventions and therapies for a wide range of nervous system injuries and impairments informed by the field of computational modeling. The conference materials can be found at https://dare2023.usc.edu/.

## Background

Neurorehabilitation is a complex process. The success of neurorehabilitation strategies depends on multiple components described by the ICF, the International Classification of Functioning, Disability, and Health [1, 2]: personal factors, body functions and structures, activity and participation levels, and environmental and social support systems. The vision of neurorehabilitation is to deliver the right treatment to the right patient at the right time [3]. Even for the most effective treatments, implementation would be highly specific to each patient who needs them at the correct time, frequency, and dose to restore or maintain function. Given the complexities of the human brain, the body it controls, and their interactions with the environment, methodologies that can manage these systems, and their heterogeneities after disabling neurological injury or disease are needed. Novel and ubiquitous sensor technologies now allow for the collection of rapidly expanding amounts of physiological and behavioral data. Increasing computational power and efficiencies further contribute to unprecedented research opportunities to develop innovative computational approaches and models capable of addressing these complexities. Together, these evolving challenges of data and modeling complexity have led to the emerging field of computational neurorehabilitation [4, 5]. This new field integrates computational approaches into neurorehabilitation to reveal important insights into neural control of human behaviors [6–8] and movement [9, 10] associated with recovery or rehabilitation.

The March 2023 Disability and Rehabilitation Engineering (DARE) Conference [11], co-sponsored by the National Science Foundation’s (NSF) Directorate for Engineering and the National Institutes of Health’s (NIH) National Institute of Child Health and Human Development’s (NICHD) National Center for Medical Rehabilitation Research (NCMRR), brought together researchers, clinicians, engineers, and trainees to discuss and explore new developments and opportunities in computational neurorehabilitation. Informed and inspired by the conference, program staff from NSF and NIH took the opportunity to write this commentary for this special issue. This commentary highlights innovative computational approaches with potential roles in (1) understanding the complex mechanisms underlying neurorehabilitation, (2) improving the delivery and implementation of rehabilitation care, and (3) empowering future rehabilitation workforce. The authors reflect on currently supported efforts by both agencies in promoting research that creates or uses computational approaches. The authors also highlight the specific and complementary programmatic opportunities from both agencies in computational neurorehabilitation as an integral part of the nation’s future healthcare system.

### Using computational models to understand complex mechanisms in neurorehabilitation

Computational modeling is often considered the “third paradigm” of scientific discovery [12], alongside theory and experiments. Decades of NSF and NIH supported research (“Alignment with federal agencies” section) have demonstrated that combining theoretical and physics-based models can enhance the rigor of experimental design and lead to a deeper understanding of complex human systems. This has also been demonstrated in neurorehabilitation research, where mechanistic models have revealed the underlying principles and dynamics of a motor recovery system [13, 14] while physics-based models provide a more detailed description of the physical interactions and properties of neuromuscular and musculoskeletal systems [15]. Both mechanistic and physics-based models can contribute to new theoretical frameworks. By combining these two approaches, researchers can develop more accurate and comprehensive models that capture aspects of these systems that are often inaccessible in human subjects research. This combination of mechanistic and physics-based approaches has the potential to lead to discovery of previously unknown but potentially critical variables and relationships. The combination may also provide insight into the underlying neural basis of recovery at the systems and behavioral levels. Ultimately, these model-based frameworks may allow for new, mechanistic experimental designs to conduct both hypothesis-driven human neuroscience studies of brain and behavior and clinical trials of newly developed treatment strategies.

### Using computational models to improve delivery and implementation of rehabilitation care

**Fig. 1:**
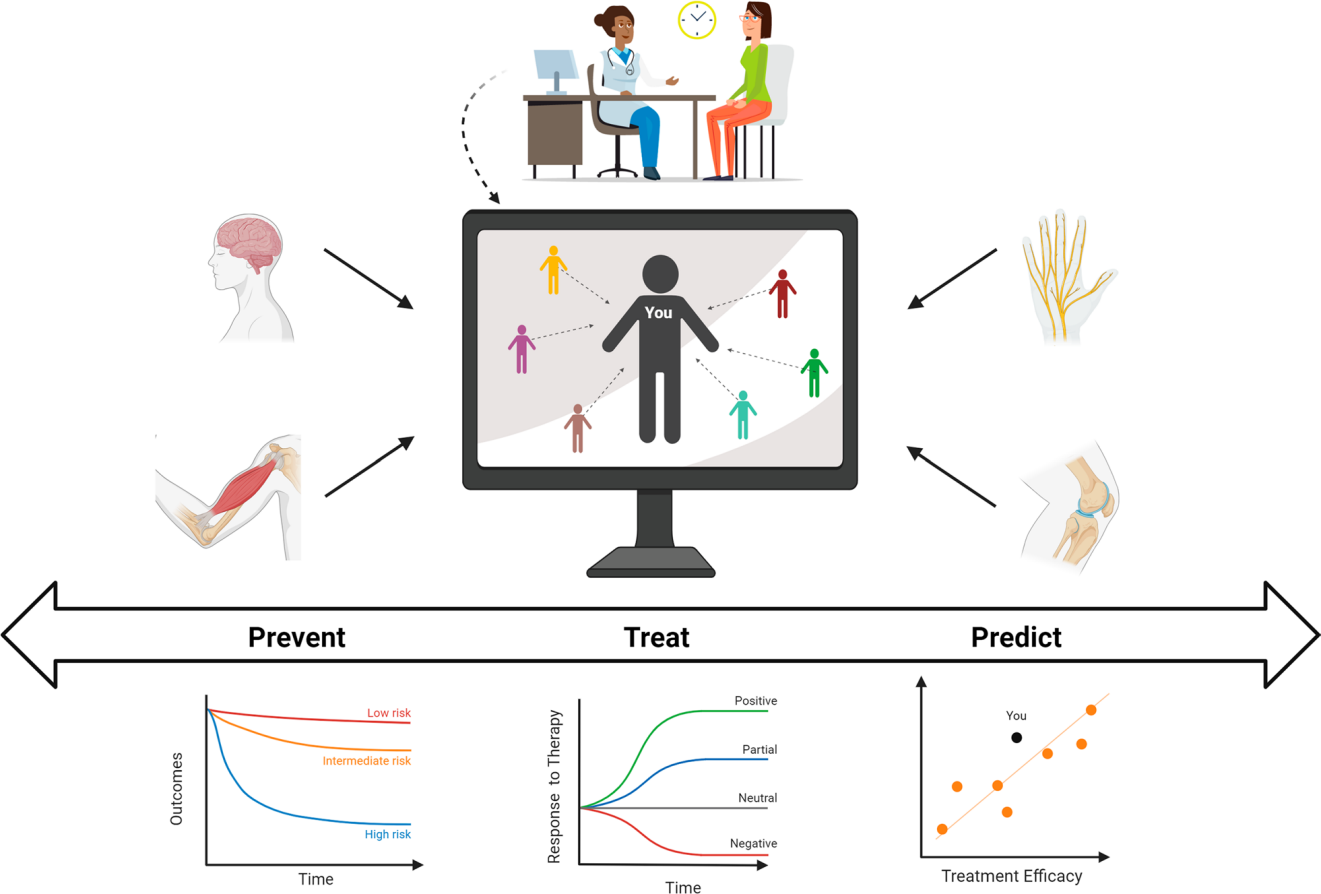
The future of neurorehabilitation. In discussions with your clinician about your health, access to your personal baseline data of your cognition, physicality, biology, and more will be readily assessable. Shared data from de-identified patients with similar clinical phenotypes allows the clinician to compare your health against that of your population. Your data could then be modeled to provide preventive care guidance, efficacious treatment plans, and allow for the experimentation of novel hypotheses that may predict outcomes beyond the feasibility of human subjects research. Figure designed in BioRender

With the emergence of ubiquitous, multi-modal sensors, the ability to capture the relative complexities of human behaviors in quantifiable parameters: cognition, perception, motor control, and emotion, to name a few, is in reach. These parameters can be further merged with social and environmental determinants of health [16] to create a complete picture of a person’s function. For artificial-intelligence-enabled biomedicine, these data could be used to create digital twins of patients and the environment associated with their care (Fig. 1). The definition of a digital twin was recently modified to include “a set of virtual information constructs that mimics the structure, context, and behavior of a natural, engineered, or social system (or system-of-systems), is dynamically updated with data from its physical twin, has a predictive capability, and informs decisions that realize value. The bidirectional interaction between the virtual and the physical is central to the digital twin” [17]. In healthcare, digital twins could be used to create personalized models of patients that allow clinicians to identify the most effective approach to optimize healthcare delivery [18]. These in silico systems would combine data such as muscle strength, joint range-of-motion, attention, and functional brain connectivity, and data gathered from similar patient populations, to identify the most effective, efficient, and economical rehabilitation strategies by simulating different scenarios. Note, however, that digital twins are not without challenges [17]. Table 1 summarizes key issues in developing a digital twin for healthcare that must be overcome as presented at a National Academies of Sciences, Engineering, and Medicine Workshop in January 2023 [18].

**Table 1:** Challenges identified in creating a digital twin for medicine

(1) Identify and solve difficult scientific problems that arise at different scales (e.g., systems biology, biophysics, the immune system)
(2) Address gaps in modeling (e.g., multiscale hybrid stochastic models, model design that facilitates updates and expansion, reusable models, and model standards)
(3) Develop appropriate collection modalities for patient data (e.g., noninvasive technologies and imaging capabilities)
(4) Develop novel forecasting methods (i.e., learning from successful hurricane forecasting)
(5) Develop data analytics methods for model recalibration from patient measurements
(6) Train a highly educated workforce
(7) Create appropriate funding models for individual medical digital twin projects from conception to prototype, and for larger infrastructure development projects

By using computational models, researchers and clinicians may be able to predict the effects of different training protocols on neural function, optimize the timing and intensity of rehabilitation interventions, and personalize treatment plans based on individual patient characteristics [5]. Moreover, monitoring patient progress over time allows clinicians to develop adaptive interventions that can adjust in real time to changes in patient behavior or response to treatment. Similar concepts have been raised in the literature in the context of adaptive behavior and plasticity [19]. Continual, real-time monitoring and assessments can lead to more effective and efficient rehabilitation, reducing the time and resources required to achieve meaningful outcomes. These efficiencies are increasingly important as hospital stays for patients with traumatic neurological injuries and stroke in the United States continue to shorten [20], thereby accelerating the timing for early in-home rehabilitation need and support. As more rehabilitation takes place at home, the need to monitor and deliver care remotely (telerehabilitation) continues to increase since the COVID-19 pandemic [21].

Beyond predicting how patients respond to standard therapies, models could simulate how a heterogeneous patient population would respond to an experimental treatment by iterating therapy dose, timing, procedure, etc., on as many virtual subjects as necessary to provide a sound evaluation. As an example, cardiac in silico models have been shown to be able to evaluate drug trials to predict clinical risk of drug-induced arrhythmias with 89% accuracy [22]. In comparison to the cardiac system, much about the human brain and its disease etiologies remains largely unknown; a computational approach could potentially play a significant role in dealing with such complexity and heterogeneity. Ultimately, computational models could expedite research and breach boundaries and enable approaches that are currently unfathomable.

### Using computational models in neurorehabilitation requires an interdisciplinary workforce

Computational modeling serves as a platform to bring rehabilitation-invested professionals from various disciplines into an integrated patient-centered ecosystem. Transformative and innovative solutions for rehabilitation arise from a convergence of expertise from clinicians, scientists, statisticians, engineers, patients, and care partners. The clinical team (i.e., physicians, nurses, and therapists) plays a critical role in delivering interventions (often behavioral or non-pharmacological) and providing the associated patient care. In addition to their clinical experience and skills, clinicians can partner with engineers, scientists, and statisticians to develop the best tools and technologies to inform and perform their therapies. Engineers bring expertise in novel sensor design, measurement, computational models and methods, as well as rehabilitation and assistive devices that can enhance clinical practice and empower patients. Lastly, persons with lived experience (i.e., patients and caregivers) can be tapped to provide insights as to meaningful outcome measures and accessibility of therapies. In turn, the resulting data from these insights can be fed back into the ecosystem for further computation. One can imagine a framework in which each of these groups contributes to a robust and synergistic learning health system.

## Gaps and opportunities

NIH and NSF have funded decades of research on computational modeling and neurorehabilitation. All NIH funded projects can be accessed at the NIH Reporter (via https://reporter.nih.gov/) while NSF projects can be accessed at the NSF Award Search (via https://www.nsf.gov/awardsearch/). In spite of decades of funding, the basic principles for how the human brain controls behaviors, including its role in neurorehabilitation, have yet to be discovered. As scientists and engineers across multiple disciplines continue to address this scientific gap, the authors have identified near-term opportunities for further research. For computational models to be adopted into clinical practice, they need to be seamlessly integrated into the clinical workflow and connected with clinical outcome measures. Many neurorehabilitation outcome measures are based on patient self-report or clinician assessments. Some outcome measures do not capture the full complexity of the behavior being evaluated or may not be sensitive to subtle changes in function or quality of life. These limitations present opportunities that the field can address. Researchers can bridge engineering figures of merit (FoM) with domain-specific and performance-based clinical measures (e.g., described in [23]). Common engineering FoM (e.g., latency, accuracy, signal-to-noise ratio, spatial and/or temporal resolution, etc.) may not easily map to clinical measures and some engineering solutions may not be connected to actual patient or clinician priorities. Interdisciplinary teams can work through these challenges together to improve patient care.

To grow the field of computational modeling in neurorehabilitation, trust must be earned through rigorous validation and open data. With regards to rigor, two types of model validation should be considered: engineering perspective and patient experience. From an engineering perspective, several challenges remain in model validation [24, 25]. Base models are often not representative of the patient population including gender, age, race, and impairment [26]. Many validation studies are conducted with electromyography sensors, which often only provide a coarse measure of muscle activation patterns. With few exceptions [27, 28], there is a paucity of noninvasive sensors that can measure the in vivo internal forces experienced by muscles and tendons before and after treatment. This fundamentally limits the accuracy of model validation. From the patient’s perspective, one can assume that most models will predict the maximum achievable recovery for a patient, or capacity. It is important, however, to receive feedback from patients on whether they are experiencing functional recovery consistent with model prediction and in domains most important to the patient (e.g., time available for therapy). It is imperative that patient input be captured so that deviations from predictions could be learned by the model to improve future treatment in a continual manner over the lifespan of the patient. The model should be flexible enough to accommodate advances in clinical outcome measures. With regards to open data, access to extensive and rich, representative datasets that are ethically collected are essential for models to comprehensively test theories or principles while mitigating bias. As an emerging field, computational neurorehabilitation needs more data that is openly accessible. Taking musculoskeletal modeling as an example: a rich dataset, in the upper extremity, would significantly facilitate developing frameworks to understand when and what to personalize computationally to optimize clinical utility. Although tremendous progress has been made in lower limb musculoskeletal models [15], they too remain limited in identifying or incorporating clinically meaningful outcomes, such as minimizing pain and improving function in a lasting and sustainable way. A stronger culture of data sharing would enable researchers to compare their findings and corroborate their models. The availability of open, robust, and trusted datasets mitigates the difficulties of recruiting subjects and allows researchers to build predictive models based on more representative samples of the population.

To ensure that data is collected and stored in a consistent and interoperable manner, the findable, accessible, interoperable, and reusable (FAIR) principles [29, 30] of data sharing should be adopted by the field. Standards define the structure, format, and semantics of data, as well as the methods for data exchange and processing. Without standards, data can be inconsistent, incomplete, or incompatible, which can hinder the ability to make accurate and timely decisions based on the data. In healthcare, data standards are particularly important for ensuring that patient data is accurately and securely transmitted between healthcare providers, researchers, and other stakeholders. Furthermore, data standards can facilitate the integration of data from different sources, such as electronic health records, medical imaging systems, wearables, human machine interface (HMI), and robotics, which can provide a more comprehensive view of patient health and enable more effective healthcare delivery. While there are efforts underway to create data standards for some neurorehabilitation data, there are many opportunities for alignment in the field. Both NSF and NIH support wide sharing of all scientific data in publicly accessible data archives to further encourage collaboration and knowledge growth among researchers [31–33].

Finally, more fundamental research is essential for developing unifying conceptual frameworks, theories, and computational models [34], which must incorporate the complexities of both the nervous and musculoskeletal systems. There are multiple questions to be addressed: how will temporal changes that represent evolving computational parameters be incorporated? How will plasticity in musculoskeletal tissues and the nervous system be modeled to reflect changes in altered activity, adaptive biomechanical supports, or other outcomes of therapies? Future research may benefit from incorporating causal inference methods into neuroimaging and brain mapping techniques [35] or new directions in reinforcement learning models [36]. A benefit of this approach is that it would allow clinicians to computationally bridge knowledge across levels within the ICF framework and across timescales (i.e., point of intervention to long-term functional outcomes). In addition, hybrid approaches that combine and integrate decades of physiological knowledge with emerging machine learning algorithms [37] could create data-efficient computational models. The current state-of-the-art musculoskeletal models have yet to sufficiently incorporate components of the nervous system that are needed to study the intercausal relationship between pain, movement, and muscle control [38]. Given the complexity of the biotic (e.g., nervous system) and abiotic (e.g., electrodes, HMI) interactions, one can argue that theory-driven mechanistic models are an essential complement to data-driven computational models in this domain.

In summary, the increasingly vast and varied datasets to be generated by neurorehabilitation research will require effective data sharing based on FAIR principles, new theory-driven computational models combining neuroscience [34] and biomechanics [39], and the development of new engineering figures of merit that relate to clinical outcome measures [40] that will collectively present many new and exciting research opportunities.

## Alignment with federal agencies

Computational approaches are an emerging force that could shape the future of biomedicine and healthcare. Joint NSF-NIH initiatives that have promoted computational research relevant to neurorehabilitation include the Brain Research Through Advancing Innovative Neurotechnologies (BRAIN®) Initiative [41, 42], the Collaborative Research in Computational Neuroscience (CRCNS) program, and the Smart Health and Biomedical Research in the Era of Artificial Intelligence and Advanced Data Science (SCH) program. These joint funding programs are particularly well suited for advancing the opportunities identified in this perspective.

In addition to joint agency partnerships, the NSF offers several funding opportunities to support rehabilitation engineering research. The DARE program is one of the few congressionally mandated programs at the NSF exclusively created to generate engineering knowledge to improve the quality of life of persons with disabilities, including visible and invisible disabilities. Funding by the DARE program focuses on high-risk/high-reward foundational engineering research that has future translational applications through new technologies (including HMI), devices, or software; advancing knowledge of normal or pathological human motion; or understanding injury mechanisms. While the NSF’s Directorate for Engineering supports projects that include human subjects or appropriate animal models, NSF cannot support clinical trial research. This is an important consideration for investigators as metrics for success will be based on engineering figures of merits and optionally clinical outcomes, since interventions based on clinical outcome measures are outside the purview of the NSF. Complimentary to the DARE program are the Engineering of Biomedical Systems (EBMS) program that supports fundamental and transformative research that integrates engineering and life sciences to solve biomedical problems, the Biomechanics and Mechanobiology (BMMB) program that supports fundamental research on biological mechanics across multiple scales—from subcellular to whole organism, and the Mind, Machine and Motor Nexus (M3X) program that supports research on the reciprocal interactions—mediated by motor manipulation—between human cognition and embodied and intelligent engineered systems (e.g., including HMI and robotics). Likewise, there are collaborative programs within the NSF that cross directorates where computational rehabilitation may fit such as Cyber-Physical Systems (CPS), Foundational Research in Robotics (FRR), Integrative Strategies for Understanding Nneural and Cognitive Systems (NCS), Cyberinfrastructure for Sustained Scientific Innovations (CSSI), and Computational and Data-Enabled Science and Engineering (CDS&E). For researchers interested in developing novel, energy-efficient, non-invasive sensor technologies appropriate for real world data collection and model validation, the Biophotonics, Biosensing, and Communications, Circuits, and Sensing-Systems (CCSS) programs may be in scope. Further details of the NSF’s funding opportunities for research related to rehabilitation can be found here: https://www.nsf.gov/eng/rehab.jsp.

Alternatively, the NSF has topic competitions announced via the Dear Colleague Letter (DCL) and the Request for Information (RFI) mechanisms that have the potential to focus on computational rehabilitation. Since 2007, the Emerging Frontiers in Research and Innovation (EFRI) program [43] has solicited input from the community every 2 years for their ideas on transformative opportunities that would lead to new areas for fundamental or applied research, new industries or capabilities that result in a leadership position for the United States and/or significant progress on a recognized national need or grand challenge. Additionally, since 2019, the Convergence Accelerator (CA) program [44] annually gathers ideas from the community and then, based on these ideas, hosts workshops on use-inspired applications fed by basic science and discovery already performed by other NSF directorates. Ultimately, both the EFRI and CA programs release solicitations as funding opportunities for the rigorously vetted topics of interest during their respective cycles. Thus, these are opportunities for the computational neurorehabilitation community to heighten the importance of and accelerate the translation of the field.

The NIH has identified several priorities related to computational modeling for neurorehabilitation, which aim to accelerate the development and adoption of innovative technologies to improve rehabilitation outcomes. These priorities include advancing the development and validation of computational models for predicting the effects of interventions on brain function and behavior, integrating multiple sources of data, including biological, behavioral, and environmental data, to provide a more comprehensive understanding of the factors that influence recovery from neurological injury, and developing new technologies, such as virtual reality, brain-computer interfaces, and mobile health apps, to enhance the effectiveness and accessibility of rehabilitation interventions. Additionally, the NIH recognizes the urgent need to develop new methods for data analysis and sharing, to promote the integration of data from different sources and enable collaboration across disciplines and institutions. By focusing on these priorities, the NIH hopes to accelerate the applications of the computational approach in neurorehabilitation research and ultimately to enhance evidence-based and knowledge-informed clinical practice. These goals are cross-cutting and highly relevant to multiple NIH Institutes and Centers, especially the missions of the NCMRR [45] (located within the NICHD) and the National Institute of Neurological Disorders and Stroke (NINDS) [46]. Applicants are encouraged to submit applications through the investigator-initiated notice of funding opportunities.

## Conclusion

Computational models are powerful tools for understanding complex systems like the human brain–body system and how it is impacted by neurological injury and disease. Computational modeling has great potential to improve neurorehabilitation care through insights gained about the underlying mechanisms and dynamics of recovery in patients. Computational models can also integrate large amounts of data from multiple sources, providing a comprehensive and holistic view of the patient during neurorehabilitation. This can lead to new discoveries and insights that would be difficult or impossible to obtain through experimental or observational approaches alone, though there are challenges to overcome before it is translated into the clinic. Overall, data science and computational approaches to neurorehabilitation constitute emerging opportunities for exciting new research areas. For the benefit of the United States and the world, the NSF advances the creation of new engineering knowledge enabled by new computational capabilities while the NIH advances the development of new interventions and therapies for a wide range of brain injuries and conditions informed by the field of computational modeling.

## Data Availability

Not applicable.

## Abbreviations

BRAIN^®^: Brain Research Through Advancing Innovative Neurotechnologies Initiative
BMMB: Biomechanics and Mechanobiology
CA: Convergence Accelerator
CCSS: Communications, Circuits, and Sensing-Systems
CRCNS: Collaborative Research in Computational Neuroscience
CDS&E: Computational and Data-Enabled Science and Engineering
CPS: Cyber-Physical Systems
CSSI: Cyberinfrastructure for Sustained Scientific Innovations
DCL: Dear Colleague Letter
DARE: Disability and Rehabilitation Engineering
EBMS: Engineering of Biomedical Systems
EFRI: Emerging Frontiers in Research and Innovation
FAIR: Findable, accessible, interoperable, and reusable
FoM: Figures of merit
FRR: Foundational Research in Robotics
HMI: Human machine interface
ICF: International Classification of Functioning, Disability and Health
M3X: Mind, Machine and Motor Nexus
NCS: Integrative Strategies for Understanding Neural and Cognitive Systems
NCMRR: National Center for Medical Rehabilitation Research
NICHD: National Institute of Child Health and Human Development
NIH: National Institutes of Health
NINDS: National Institute of Neurological Disorders and Stroke
NSF: National Science Foundation
RFI: Request for Information
SCH: Smart Health and Biomedical Research in the Era of Artificial Intelligence and Advanced Data Science

## Declarations

This material is based on work supported by (while serving at) the National Science Foundation. Any opinions, findings, conclusions, or recommendations expressed in this material are those of the authors and do not necessarily reflect the views of the National Science Foundation. The opinions expressed in this article are the author's own and do not reflect the view of the National Institutes of Health, the Department of Health and Human Services, or the United States Government.
